# Editorial: Endocrine disruptors: mechanism of action and implications for human health

**DOI:** 10.3389/fcell.2024.1378345

**Published:** 2024-02-07

**Authors:** Giulia Gaggi, Federica Barbagallo, Luca De Toni, Ines Bucci

**Affiliations:** ^1^ Reprogramming and Cell Differentiation Lab, Center for Advanced Studies and Technology (CAST), Chieti, Italy; ^2^ Department of Medicine and Aging Sciences, “G. D’Annunzio” University of Chieti-Pescara, Chieti, Italy; ^3^ UdA TechLab Center (UdATech), Chieti, Italy; ^4^ Faculty of Medicine and Surgery, “Kore” University of Enna, Enna, Italy; ^5^ Department of Medicine, Unit of Andrology and Reproductive Medicine, University of Padova, Padova, Italy

**Keywords:** endocrine disruptors, infertility, endocrinology, development, reproduction

Endocrine disruptors (EDs) represent a heterogeneous group of natural and chemical compounds capable of interfering with the delicate balance of the endocrine system (Gaggi et al., 2023a), which regulates various physiological processes through the secretion of hormones. The disarrangement of hormonal pathways through exposure to EDs can have profound consequences on human health following early embryo development (Di Credico et al., 2023a; Gaggi et al., 2023b). Accordingly, the ubiquitous presence of EDs in everyday products such as pesticides, plastics, and personal care items mimic or block the action of natural hormones and is associated with reproductive problems, developmental abnormalities, and an increased risk of certain diseases (Di Credico et al., 2023b; Ullah et al., 2023).

One of the significant challenges posed by EDs is their ability to exert adverse effects at incredibly low concentrations, making them difficult to regulate effectively. The widespread use of these substances in everyday items further complicates the task of minimizing exposure (Vandenberg, 2014). As researchers delve into the long-term consequences of exposure to EDs, the imperative to address and mitigate their presence in the environment becomes increasingly evident. Despite recent advances in understanding the mechanisms behind the impact of EDs on human life, there remains a need to comprehensively disclose the fundamental biological processes through which these disruptors exert their influence.

The studies presented in this Research Topic aim to shed light on the multifaceted impact of endocrine disruptors on human health. Researchers have explored various aspects, ranging from the effects of estrogen-mimicking compounds in cosmetics to the interaction of EDs with the androgen receptor signaling pathway, elucidating also the impact of natural phenomenon and chemical substances on fertility (De Toni et al.; Pappalardo et al.; Sheikh et al.; Rispo et al.).

Rispo et al. extensively reviewed the effects of estrogen-mimicking compounds commonly found in cosmetics. While these compounds can have positive effects such as preventing skin damage and aging, the study highlights also their darker side. The authors reported a link between these compounds and premature female sexual development and gynecomastia in males. The interaction with both estrogen receptors alpha and beta (ERα, β) underscores the complexity of the impact of these compounds on human health.

Sheikh et al. explored the interaction of EDs with the androgen receptor (AR) signaling pathway, focusing on two common pyrethroids: cypermethrin and deltamethrin. The study demonstrated that these substances are associated with impaired male reproductive function. Using advanced computational techniques, the authors revealed a significant overlap between cypermethrin, deltamethrin, and AR native ligands such as testosterone, highlighting the possible interference of these two EDs with the AR signaling pathway, potentially leading to infertility.

Pappalardo et al. delved into the impact of heavy metals, particularly cadmium (Cd), on male reproductive function. Cd, prevalent in the soil and agricultural products, can enter the human body through the food chain or by lifestyle habits such as smoking. The study exposed human spermatozoa to Cd *in vitro*, revealing a reduction in the total motile sperm fraction compared to non-exposed controls. This finding suggests a potential reduction in male fertility associated with exposure to heavy metals.

Finally, De Toni et al. explored the impact of the environmental phenomenon of global warming on testis function. The study highlighted the potential consequences of global warming-induced heat stress, leading to oxidative stress, DNA damage, apoptosis in testicular cell, and a reduction in serum testosterone levels. These effects collectively contribute to impairing testicular function and reducing sperm quality.

This Research Topic contributes to underscoring the intricate and pervasive nature of their impact on human health. From estrogen-mimicking compounds in cosmetics to the disruptive influence on androgen receptor signaling pathways and the adverse effects of heavy metals and global warming, the findings emphasize the urgent need for regulatory measures and mitigation strategies. As we continue to unravel the complexities of endocrine disruption, the proper address of these issues becomes of paramount importance to safeguard human health and to maintain the ecological balance.
